# Characterizing *Salmonella enterica* Serovar Choleraesuis, var. Kunzendorf: A Comparative Case Study

**DOI:** 10.3389/fvets.2019.00316

**Published:** 2019-09-20

**Authors:** Alessandra Longo, Sara Petrin, Eleonora Mastrorilli, Alessia Tiengo, Antonia Anna Lettini, Lisa Barco, Antonia Ricci, Carmen Losasso, Veronica Cibin

**Affiliations:** World Organisation for Animal Health (OIE), National Reference Laboratory for Salmonellosis, Istituto Zooprofilattico Sperimentale delle Venezie, viale dell'Università, Legnaro, Italy

**Keywords:** *Salmonella* serotyping, antigenic *formula*, serovar Choleraesuis, biotype, xMAP^®^*Salmonella* serotyping assay, whole genome sequencing, MLST

## Abstract

Different *Salmonella* serovars generally display different antigenic *formulae*, but there are some exceptions. For instance, the same antigenic *formula*, 6,7:c:1,5, is shared by *Salmonella enterica* serovar, Paratyphi C, Typhisuis, and Choleraesuis. Moreover, three biotypes have been described within the *S*. Choleraesuis serovar. A distinction among such biotypes can only be based on biochemical behaviors (biotyping) posing serious concerns when rapid characterization is required. The study of an outbreak of severe epizootic salmonellosis in wild boars occurred in Italy between 2012 and 2014 and the typing of the isolates recovered from the outbreak were used to test different approaches for serovar identification. A number of 30 *S*. Choleraesuis var. Kunzendorf isolates from the outbreak were typed by means of four different methods to derive serovar and biotype: (i) slide agglutination method followed by biochemical tests, (ii) suspension array xMAP^®^ *Salmonella* Serotyping Assay (SSA), (iii) whole genome sequencing (WGS) and data analysis using SeqSero tool, and (iv) WGS and data analysis using *Salmonella* TypeFinder tool. Slide agglutination, xMAP^®^ SSA and WGS, followed by SeqSero analysis, are methods that infer the serovars according to the White-Kauffmann-Le Minor (WKL) scheme, based exclusively on antigens. Using these methods, isolates with incomplete antigenic *formulae* could be misleadingly excluded from an outbreak. On the contrary, WGS followed by *Salmonella* TypeFinder data analysis, which predicts the serotype on the basis of Multilocus sequence typing (MLST), might be able to cluster together isolates belonging to the same outbreak irrespective of the antigenic *formula*. Results suggest the benefit of routine use of a combination of *in silico* MLST and antigenic *formula* analysis to solve specific ambiguous case studies for outbreak investigation purposes.

## Introduction

The White-Kauffmann-Le Minor (WKL) scheme summarizes antigenic *formulae* of all known *Salmonella* serovars, on the basis of antigenic variability in the outer membrane lipopolysaccharides (O antigen), flagellar proteins (H1 and H2 antigens) and capsular polysaccharide (Vi antigen) (1). The most recent edition of the WKL scheme has identified over 2,500 serovars belonging to the five subspecies of *Salmonella enterica* (1, 2).

Traditional serotyping of *Salmonella* based on slide agglutination has been used for decades worldwide (3), and it is still considered the gold standard method for *Salmonella* serotyping. According to this phenotype-based approach, the surface antigens are detected by agglutination of bacterial cells using specific *Salmonella* antisera (3). Traditional serotyping is labor intensive, and it requires trained technicians to provide valuable data (3). Another limitation of this method, which leads to inconclusive results, is a possible loss of expression of antigens required for definitive serovar identification (for example rough strains) (4). For all these reasons, molecular methods for *Salmonella* serotyping have been developed (3). An example of molecular alternative methods for *Salmonella* serotyping is a multiplex bead-based suspension array developed to detect the most common serovars using Luminex technology (5). Moreover, the technological advancements of Whole Genome Sequencing (WGS) and the improved bioinformatic analyses are revolutionizing surveillance programs and WGS data could also be used to derive information about *Salmonella* characteristics, such as serotype antimicrobial resistance determinants, virulence genetic factors, plasmid types, and *in silico* Multi Locus Sequence Type (MLST) (6).

Different *Salmonella* serovars generally display different antigenic *formulae*, but there are also some exceptions. Historically, different names have been assigned to serovars showing the same antigenic *formula* but differing either by biochemical characteristics, pathogenicity, or habitats (1).

The antigenic *formula* 6,7:c:1,5 is shared by different serovar: Paratyphi C, Typhisuis, Choleraesuis (1). Furthermore, three biotyping subdivisions on the basis of H_2_S production and the utilization of mucate and dulcitol have been described within Choleraesuis serovar: Choleraesuis *sensu stricto*, Choleraesuis var. Kunzendorf, and Choleraesuis var. Decatur (7).

Serovar Paratyphi C is associated with enteric fever in humans; serovar Typhisuis is associated with chronic paratyphoid/caseous lymphadenitis in swine (8) and serovar Choleraesuis may cause serious outbreaks of salmonellosis and paratyphoid in pigs (9), with clinical outcomes, such as enterocolitis and septicemia (10), often resulting in fatal systemic disease (11). This serovar is currently highly prevalent in North America and Asia, but it is rare in Australia and the European Union (EU) (12).

An unexpected and sudden outbreak of severe epizootic salmonellosis due to *S*. Choleraesuis occurred in wild boars in Italy between 2012 and 2014; recovered isolates were typed for outbreak investigation purposes (13). A total of 30 isolates belonging to the outbreak were serotyped using the slide agglutination method, and biochemical tests were performed to identify the biotype. Where serotyping didn't work, additional tests were used to determine the serotype.

Results suggest the benefit of a combination of *in silico* MLST and antigenic *formula* detection to deep insight into a specific case of uncertainty in *Salmonella* serovar attribution.

## Materials and Methods

### Bacterial Isolates

A panel of thirty *Salmonella* wild boar isolates was included in this study (Table 1). All the isolates were collected by the Istituto Zooprofilattico Sperimentale delle Venezie, Legnaro, Italy between 2012 and 2014. Isolation was performed according to ISO 6579:2002/Amd 1: 2007.

**Table 1:** Entire panel of analyzed isolates and serotyping results using different approaches: phenotypic serotyping and biochemical tests, xMAP^®^ *Salmonella* Serotyping Assay (SSA), WGS (data analysis with SeqSero tool) and WGS (data analysis with *Salmonella* TypeFinder tool).

**ID**	**Origin**	**Isolation year**	**Phenotypic serotyping/biochemical tests**	**xMAP SSA**	**WGS (Seq Sero)**	**WGS (*Salmonella* TypeFinder)**
12/54912	WB	2012	*S*. enterica subsp. enterica (Gr. C1 - 6,7: -: 1,5)	C1:c:1,5	6,7:c:1,5	*S*.Choleraesuis var. Kunzendorf
12/100302	WB	2012	*S*. Choleraesuis var. Kunzendorf	C1:c:1,5	6,7:c:1,5	*S*.Choleraesuis var. Kunzendorf
12/164369	WB	2012	*S*. Choleraesuis var. Kunzendorf	C1:c:1,5	6,7:c:1,5	*S*.Choleraesuis var. Kunzendorf
12/118163	WB	2012	*S*. Choleraesuis var. Kunzendorf	C1:c:1,5	6,7:c:1,5	*S*.Choleraesuis var. Kunzendorf
12/95668	WB	2012	*S*. Choleraesuis var. Kunzendorf	C1:c:1,5	6,7:c:1,5	*S*.Choleraesuis var. Kunzendorf
12/167530	WB	2012	*S*. enterica subsp. enterica (-: -: 1,5)	-:c:1,5	-:c:1,5	*S*.Choleraesuis var. Kunzendorf
12/150332	WB	2012	*S*. Choleraesuis var. Kunzendorf	C1:c:1,5	6,7:c:1,5	*S*.Choleraesuis var. Kunzendorf
12/171410	WB	2012	*S*. enterica subsp. enterica (-: c: 1,5)	-:c:1,5	-:c:1,5	*S*.Choleraesuis var. Kunzendorf
12/87099	WB	2012	*S*. enterica subsp. enterica (Gr. C1 - 6,7: -: 1,5)	C1:c:1,5	6,7:c:1,5	*S*.Choleraesuis var. Kunzendorf
12/90074	WB	2012	*S*. Choleraesuis var. Kunzendorf	C1:c:1,5	6,7:c:1,5	*S*.Choleraesuis var. Kunzendorf
12/90639	WB	2012	*S*. Choleraesuis var. Kunzendorf	C1:c:1,5	6,7:c:1,5	*S*.Choleraesuis var. Kunzendorf
12/164644	WB	2012	*S*. enterica subsp. enterica (-: -: 1,5)	-:c:1,5	-:c:1,5	*S*.Choleraesuis var. Kunzendorf
12/78468	WB	2012	*S*. Choleraesuis var. Kunzendorf	C1:c:1,5	6,7:c:1,5	*S*.Choleraesuis var. Kunzendorf
12/163644	WB	2012	*S*. Choleraesuis var. Kunzendorf	C1:c:1,5	6,7:c:1,5	*S*.Choleraesuis var. Kunzendorf
12/127550	WB	2012	*S*. Choleraesuis var. Kunzendorf	C1:c:1,5	6,7:c:1,5	*S*.Choleraesuis var. Kunzendorf
12/90090	WB	2012	*S*. Choleraesuis var. Kunzendorf	C1:c:1,5	6,7:c:1,5	*S*.Choleraesuis var. Kunzendorf
12/159467	WB	2012	*S*. Choleraesuis var. Kunzendorf	C1:c:1,5	6,7:c:1,5	*S*.Choleraesuis var. Kunzendorf
13/821	WB	2013	*S*. Choleraesuis var. Kunzendorf	C1:c:1,5	6,7:c:1,5	*S*.Choleraesuis var. Kunzendorf
13/76379	WB	2013	*S*. Choleraesuis var. Kunzendorf	C1:c:1,5	6,7:c:1,5	*S*.Choleraesuis var. Kunzendorf
13/9861	WB	2013	*S*. Choleraesuis var. Kunzendorf	C1:c:1,5	6,7:c:1,5	*S*.Choleraesuis var. Kunzendorf
13/104977	WB	2013	*S*. Choleraesuis var. Kunzendorf	C1:c:1,5	6,7:c:1,5	*S*.Choleraesuis var. Kunzendorf
13/66045	WB	2013	*S*. enterica subsp. enterica (-: -: 1,5)	-:c:1,5	-:c:1,5	*S*.Choleraesuis var. Kunzendorf
14/9953	WB	2014	*S*. enterica subsp. enterica (-: -: 1,5)	-:c:1,5	-:c:1,5	*S*.Choleraesuis var. Kunzendorf
14/28309	WB	2014	*S*. Choleraesuis var. Kunzendorf	C1:c:1,5	6,7:c:1,5	*S*.Choleraesuis var. Kunzendorf
14/70644	WB	2014	*S*. Choleraesuis var. Kunzendorf	C1:c:1,5	6,7:c:1,5	*S*.Choleraesuis var. Kunzendorf
14/82778	WB	2014	*S*. Choleraesuis var. Kunzendorf	C1:c:1,5	6,7:c:1,5	*S*.Choleraesuis var. Kunzendorf
14/82812	WB	2014	*S*. Choleraesuis var. Kunzendorf	C1:c:1,5	6,7:c:1,5	*S*.Choleraesuis var. Kunzendorf
14/98709	WB	2014	*S*. Choleraesuis var. Kunzendorf	C1:c:1,5	6,7:c:1,5	*S*.Choleraesuis var. Kunzendorf
14/147295	WB	2014	*S*. Choleraesuis var. Kunzendorf	C1:c:1,5	6,7:c:1,5	*S*.Choleraesuis var. Kunzendorf
15/37298	WB	2015	*S*. Choleraesuis var. Kunzendorf	C1:c:1,5	6,7:c:1,5	*S*.Choleraesuis var. Kunzendorf

*Information about origin (WB = wild boar) and year of isolation are reported*.

### Phenotipic Serotyping and Biochemical Tests

All *Salmonella* isolates were serotyped by slide agglutination with *Salmonella* antisera (Statens Serum Institut, Copenhagen, Denmark) and serovar names assigned according to the WKL; distinction between the biotypes of *S*. Choleraesuis was performed by biochemical tests (H_2_S production, mucate and dulcitol fermentation) (1).

### xMAP^®^ *Salmonella* Serotyping Assay

*Salmonella* isolates were serotyped by xMAP^®^ *Salmonella* Serotyping Assay (SSA), Luminex Corp., Austin, TX, U.S. SSA is a molecular serotyping assay addressing a set of target genes involved in the expression of the most common *Salmonella* serotype-specific antigens (4, 5).

### Whole Genome Sequencing and Data Analysis

Genomic DNA was extracted using QIAamp DNA Mini Kit (Qiagen, Valencia, CA) and quantified with a Qubit 3.0 Fluorometer (Life Technologies, Carlsbad, CA). Libraries for sequencing were prepared using Nextera XT DNA Library Preparation Kit (Illumina, San Diego, CA). High-throughput sequencing was performed on Illumina MiSeq with 2 × 250 paired-end reads. Raw sequence data were submitted to the European Nucleotide Archive (http://www.ebi.ac.uk/ena) under accession number PRJEB27935.

Raw reads were assembled using SPAdes (version 3.9) (14), available online at the Center for Genomic Epidemiology (CGE) (www.genomicepidemiology.org). The serotyping was performed analyzing contigs with SeqSero (version 1.2) (15) and raw reads with *Salmonella* TypeFinder version 1.4 (https://cge.cbs.dtu.dk/services/SalmonellaTypeFinder/) (15–17).

## Results

Based on phenotypic serotyping and biochemical tests, 23 out of 30 isolates were shown to be *S*. Choleraesuis var. Kunzendorf by phenotypic serotyping and biochemical tests. All these isolates were assigned to the antigenic *formula* 6,7: c: 1,5 and showed the following biochemical features: dulcitol (–), H_2_S (+) and mucate (–).

Seven additional isolates displayed incomplete antigenic *formula*, in particular, two isolates did not present the first flagellar antigen (c), one isolate did not display the somatic antigen (Serogroup C1–antigens 6,7) and four isolates presented neither somatic nor the first flagellar antigens. The second flagellar antigen (1,5) was always detected (Table 1). The isolates with incomplete antigenic *formula* couldn't be definitively typed as *S*. Choleraesuis according to the traditional serotyping and were thus classified as *S. enterica subsp. enterica*.

*Salmonella* isolates were serotyped by xMAP^®^ SSA. Two out of the seven isolates harbored the entire panel of genes, which allowed to infer the complete antigenic *formula* (C1:c:1,5). Biochemical tests allowed the typing of these isolates as *S*. Choleraesuis var. Kunzendorf. The remaining five isolates did not display the genetic target of the relative somatic antigens.

On the basis of the WGS and data analysis with SeqSero tool, twenty-five isolates out of thirty presented a complete antigenic *formula* (6,7:c:1,5). The lack of somatic antigen sequence for the other five isolates was also confirmed by WGS analysis, leaving the typing incomplete.

Regarding the analysis with *Salmonella* TypeFinder tool, even though the antigenic *formulae* found with the preceding methods were confirmed for all tested isolates, it was possible to obtain only an indirect relationship for Sequence Type 145 with serovar Choleraesuis var. Kunzendorf.

All the results are reported in Table 1.

## Discussion

The wild boar epizootic mentioned in this work was caused by *S*. Choleraesuis var. Kunzendorf, which is considered the typical biotype of this serovar causing swine infections (9). The characterization of the *Salmonella* isolates responsible for wild boar's mortality provided us the opportunity to test different approaches to solve a specific ambiguous case study. The entire panel of isolates was serotyped with a phenotype-based approach at first, followed by biochemical tests. These analyses are labor intensive and quite long. Another limitation of traditional serotyping includes a possible loss of expression of one of the tested antigens (3). Seven isolates did not express one of the antigens required for serotyping, thus the result for the serovar assignment was incomplete (*Salmonella enterica* subsp. *enterica*). This would have misled the outbreak definition, as isolates from the same outbreak could have been assigned to different serovars.

Molecular serotyping methods offer a high-throughput alternative to traditional ones, which can strengthen the public health response capacity (18). In this study, the traditional serotyping was supported by three molecular approaches aiming at resolving the incomplete assignment of some isolates to a specific serovar. xMAP^®^ SSA was a faster approach than the traditional serotyping, however, alone, it was not sufficient to discriminate *S*. Choleraesuis var. Kunzendorf for the entire panel of tested isolates. The two *Salmonella* isolates, showing absence of c flagellar antigen according to the phenotypic method, resulted to be *S*. Choleraesuis var. Kunzendorf by using xMAP^®^ SSA. However, the lack of somatic antigen in the remaining five isolates was also confirmed by the xMAP^®^ SSA, indicating the absence of the relative genetic target (*rfb* gene) (4).

The reads obtained from the entire panel of isolates by WGS were analyzed with two different tools. SeqSero tool, which assigns the serovar according to the antigenic *formula*, confirmed the results obtained by means of the molecular method.

Finally, *Salmonella* TypeFinder tool, which predicts the serovar using MLST typing (17), identified both serovar and biotype of the entire panel of the analyzed isolates as *S*. Choleraesuis var. Kunzendorf.

This study demonstrated that the antigenic *formula* detection might be not conclusive to cluster together isolates belonging to the same outbreak. The combined use of MLST and antigenic *formula* allowed, therefore, allocation of the investigated isolates to the same outbreak, irrespective of the antigenic *formula*. This suggests the perspective of integration of different data, both molecular and epidemiological, to provide deep insight into outbreak characterization in the presence of typing ambiguity.

## Data Availability Statement

Raw sequence data were submitted to the European Nucleotide Archive (http://www.ebi.ac.uk/ena) under accession number PRJEB27935.

## Author Contributions

AL, SP, AAL, LB, AR, CL, and VC contributed conception and design of the study. AL, SP, EM, and AT performed the analysis. AL and CL wrote the manuscript. All authors contributed to manuscript revision, read, and approved the submitted version.

### Conflict of Interest

The authors declare that the research was conducted in the absence of any commercial or financial relationships that could be construed as a potential conflict of interest.
